# Advances in the development and application of microbial consortia for metabolic engineering

**DOI:** 10.1016/j.mec.2019.e00095

**Published:** 2019-05-20

**Authors:** Kamran Jawed, Syed Shams Yazdani, Mattheos AG. Koffas

**Affiliations:** a Microbial Engineering Group, International Centre for Genetic Engineering and Biotechnology, Aruna Asaf Ali Marg, New Delhi, 110067, India; b DBT-ICGEB Centre for Advanced Bioenergy Research, International Centre for Genetic Engineering and Biotechnology, Aruna Asaf Ali Marg, New Delhi, 110067, India; c Department of Chemical and Biological Engineering, Rensselaer Polytechnic Institute, Troy, NY, USA; d Department of Biological Sciences, Rensselaer Polytechnic Institute, Troy, NY, USA

**Keywords:** Co-cultivation, Microbiome, Microbial consortia, Microbial biosynthesis

## Abstract

Recent advances in metabolic engineering enable the production of high-value chemicals via expressing complex biosynthetic pathways in a single microbial host. However, many engineered strains suffer from poor product yields due to redox imbalance and excess metabolic burden, and require compartmentalization of the pathway for optimal function. To address this problem, significant developments have been made towards co-cultivation of more than one engineered microbial strains to distribute metabolic burden between the co-cultivation partners and improve the product yield. In this emerging approach, metabolic pathway modules can be optimized separately in suitable hosts that will then be combined to enable optimal functionality of the complete pathway. This modular approach broadens the possibilities to fine tune sophisticated production platforms and thus achieve the biosynthesis of very complex compounds.

Here, we review the different applications and the overall potential of natural and artificial co-cultivation systems in metabolic engineering in order to improve bioproduction/bioconversion. In addition to the several advantages over monocultures, major challenges and opportunities associated with co-cultivation are also discussed in this review.

## Highlights

• Benefits of using co-cultivation system in metabolic engineering.
• Existence of natural consortia and their application.
• Recent advancement in co-cultivation methodology for bioproductions.
• Challenges in implementing microbial consortia for microbial biosynthesis.

## 1 Introduction

Metabolic engineering of microorganisms enables production of chemicals via construction and optimization of different metabolic pathways. The functionality and overall conversion efficiency of the biosynthetic pathway depends on various factors including precursors, cofactor demand and optimal expression of the pathway enzymes. Problems arise however when, due to the complexity and length of the recombinant pathway, a single strain cannot cope with the pathway demand, a phenomenon commonly referred to as metabolic burden (Wu et al., 2016).

**Fig. 1:**
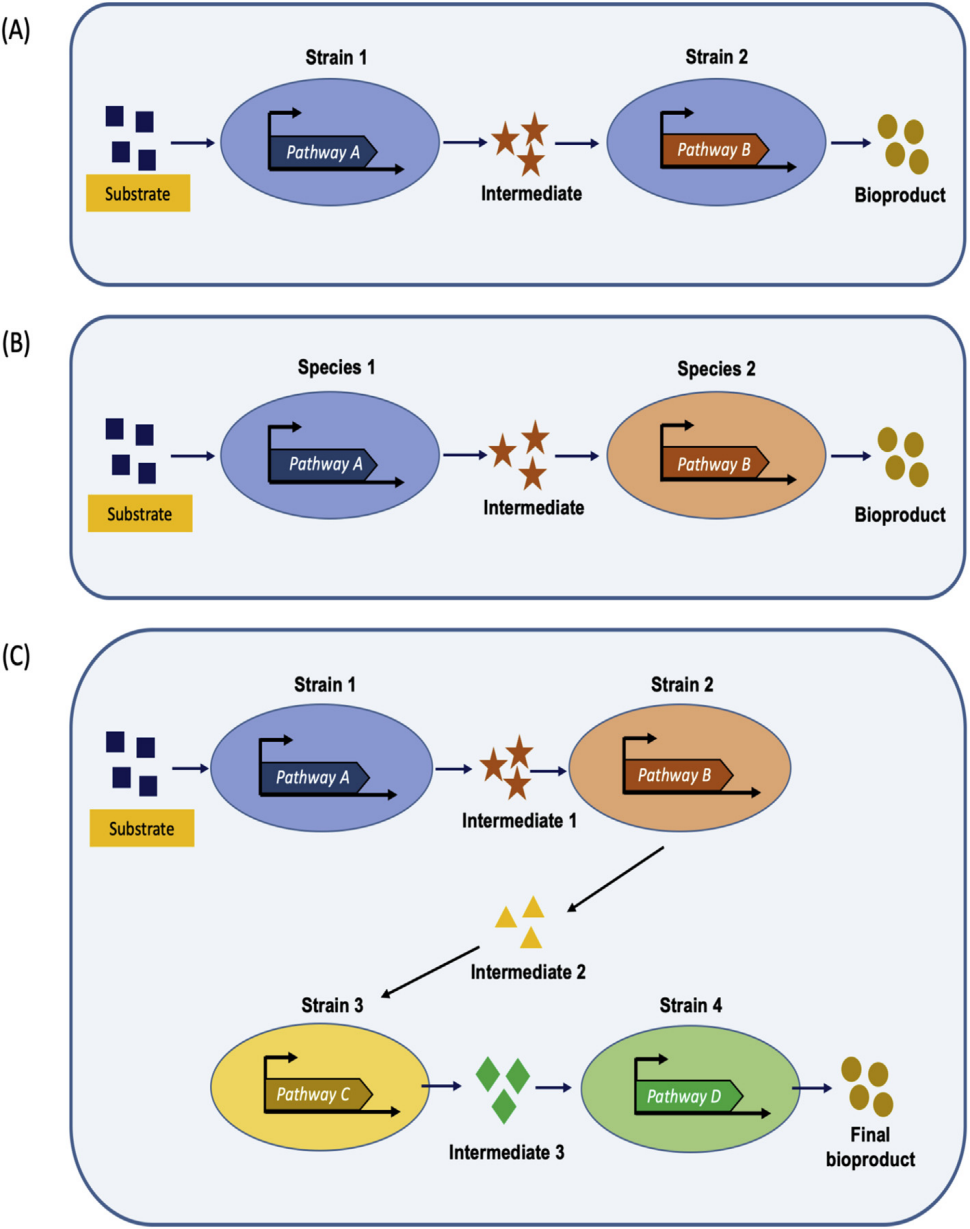
Schematic representation of artificial consortium for bioproductions. (A) Co-cultivation comprising strains of the same species, (B) Co-cultivation comprising strains from different species and (C) Co-cultivation comprising mixed strains i.e. polyculture.

To overcome the limitations posed by metabolic burden, significant developments have been made towards rationally designed microbial co-cultures to distribute metabolic burden of complex and long biosynthetic pathways into different strains/species in order to improve bioproduction performance (Jones et al., 2017, 2016; Liu et al., 2018; Saini et al., 2015; Tsoi et al., 2018; Zhang and Wang, 2016) (Fig. 1). This approach has been inspired by microbial natural consortia, which carry out complex chemical reactions to provide favourable environment for survival of the community.

The modularity in co-cultivation allows rapid optimization of the strains carrying each pathway module and latter assembly of the engineered strains into a synthetic consortium that enables optimal conversion of a substrate or precursor metabolite to the desired final product. It provides a platform to optimize each segregated pathway under optimal cellular environments for functional expression of different pathway genes. It also provides balancing of the complex pathway by optimizing the ratio of the consortia members to improve overall yield (Zhou et al., 2015). Compartmentalization between co-cultivation partners reduces the possibility of undesired cross-reactions between the pathway modules and thus enables efficient bioproduction (Martínez et al., 2016; Shong et al., 2012).

Co-cultivation methodologies are widely used in animal tissue engineering. Such approaches provide fine control of the target cells through paracrine signalling to make functional tissues (Cittadella Vigodarzere and Mantero, 2014; Paschos et al., 2015). Co-cultivations are very beneficial for testing drug efficacy during drug development as they provide more realistic in vivo–like conditions than mono-cultures. They allow high-throughput screening and in-depth monitoring of drug effects on cell–cell interactions (Fang and Eglen, 2017; Goers et al., 2014). Co-cultivation strategies have been also applied for efficient degradation of different organic contaminants (Benner et al., 2015; Mekuto et al., 2018; Zhang et al., 2013; Zhao et al., 2016).

Despite extensive work on engineering microbial consortia for chemical biosynthesis, very few co-cultivation strategies have been applied in industrial biotechnology. Such industrial applications include wastewater treatment, biogas production, and the production of traditional foods. In the case of food industry, synthetic consortia are used for making dairy products such as cheese, yoghurt and kefir; bakery products like sauerkraut and sourdough; and meat products like salami (Bader et al., 2010). Liquor industry widely uses different microbial consortia for making whisky, beer and wine (Benkerroum et al., 2005). Finally, a co-cultivation approach has extensively been used for the production of vitamin C (Guleria et al., 2016).

This review describes the recent successful implementation and applications of co-cultivation methods for microbial biosynthesis using metabolic engineering approaches. It also highlights the challenges and limitations in existing co-cultivation systems and discusses how it can be improved to reach their full potential for industrial applications.

## 2 Merits

Co-cultivation methodologies reduce and even eliminate metabolic burden of the engineered strains that typically encounter metabolic stress due to the overexpression of long and complex biosynthetic pathways in single cells. This is because functional expression of extensive metabolic pathways requires significant energy expenditure and imposes an additional drain of key precursors and co-factors and thus competes with biomass generation. Such competition often leads to impaired growth and finally poor product yield (Wu et al., 2016). Co-cultivation engineering facilitates division of metabolic labour between each constituent strain and thus has the potential to improve bioproduction and bioconversion performance (Fig. 1). The application of co-cultivation results in more significant production improvements when the functional expression of some genes of long biosynthetic pathways demands more specialized environment, when toxic intermediates are generated or when a single host is unable to meet the energy demands of energy-expensive pathways.

One of the great advantages of co-cultivation approaches is that it can involve the use of multiple species forming artificial consortia. Multiple species provide diverse environments that are best suited for optimal activity of pathway enzymes, especially enzymes that are derived from higher eukaryotes. In such a scheme, the product of one engineered strain is transported to another engineered microbe where it is further metabolized to the final product (Zhang and Wang, 2016) (Fig. 1C).

In one example, the taxadiene 5-ol biosynthesis pathway was divided between *S. cerevisiae* and *E. coli*. *E. coli* was engineered to overproduce taxadiene, while *S. cerevisiae* was used for expressing cytochrome P450s (CYPs). P450s have been notoriously challenging to express in *E. coli* despite extensive engineering efforts and, as a result, *S. cerevisiae* has traditionally been used to express these enzymes (Leonard et al., 2006). Co-cultivation of both allowed the rapid production of taxadiene in *E. coli*, which was further functionalized to taxanes by oxygenation reactions in *S. cerevisiae*. This synthetic consortium of two different microbial species was able to produce 33 mg/L oxygenated taxanes (Zhou et al., 2015).

Co-cultivation engineering offers an alternative way to avoid negative regulation of pathway intermediates on product biosynthesis yield. For example, reactive oxygen species (ROS) produced during taxane oxygenation inhibit taxadiene biosynthetic pathway enzymes (ISPG and ISPH). Spatial segregation of the pathway into two different cells provides membrane barrier to ISPG and ISPH from ROS and thus prevents inactivation (Pillai et al., 2011; Zhou et al., 2015).

**Fig. 2:**
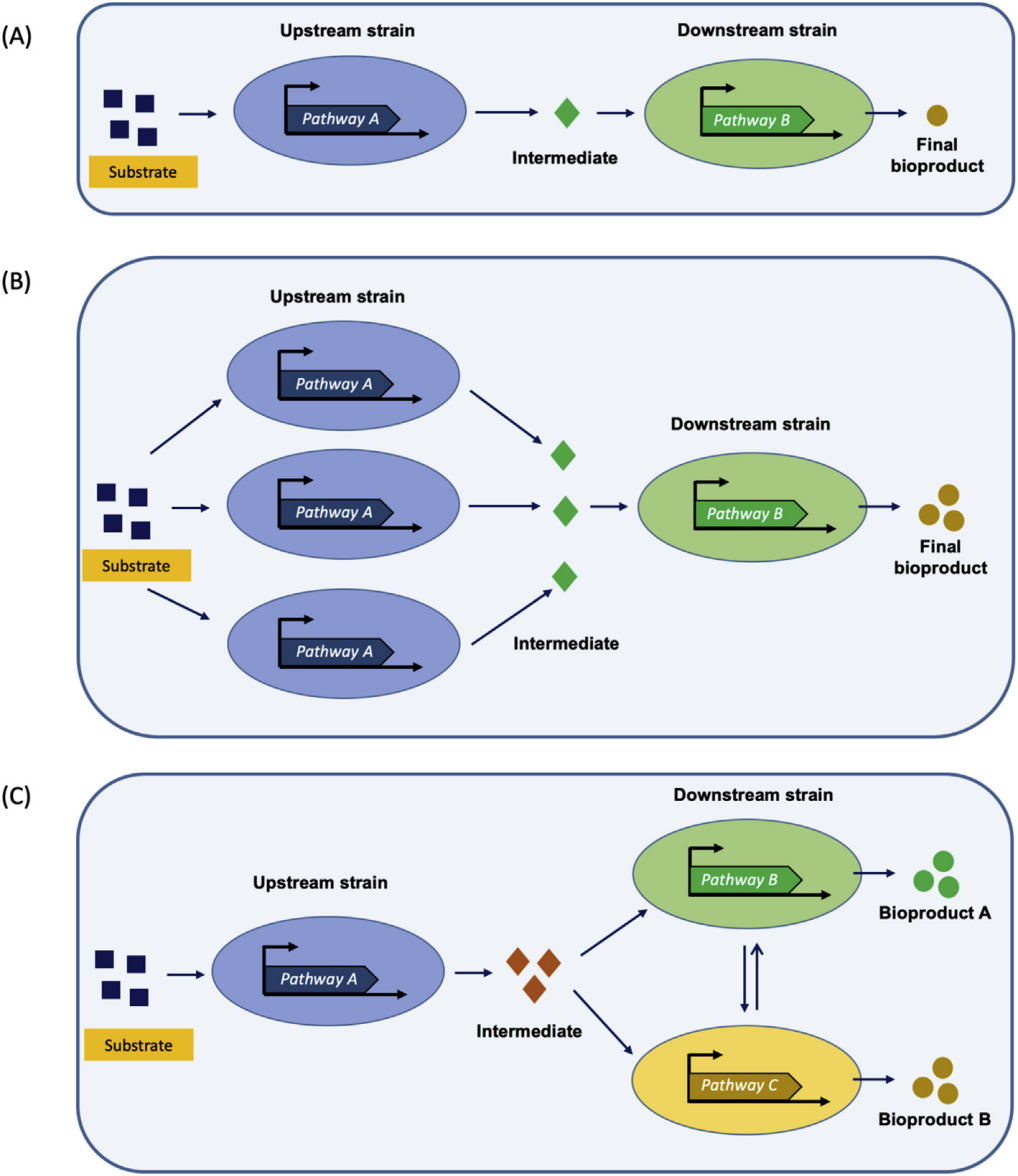
Optimization of synthetic consortium for bioproductions. (A) Equal subpopulation of each constituent strain in consortium not always yield maximum product, (B) Tuning of strain subpopulation by changing the inoculation ratio to achieve high product yield and (C) swapping of the downstream strain in a plug-and-play manner allows production of various desired products from same intermediate.

Co-cultivation also allows fine tuning of pathway modules by optimizing the relative population ratio of the synthetic consortium by changing the initial inoculation ratio (Jones and Koffas, 2016; Liu et al., 2018; Zhou et al., 2015) (Fig. 2A and B), or by inoculating a secondary strain during the cultivation of primary strain (Saini et al., 2015). Variation in population ratio changes population dynamics which leads to optimal function of each pathway module for efficient conversion of substrate to product with little or no accumulation of intermediate metabolites (Jones et al., 2016). For example, various population ratios were tested in order to improve flavonoids by changing initial inoculation cell ratios of engineered *E. coli* during co-cultivation. The maximum titer was achieved from the consortium having initial inoculation ratio of 8:2 (upstream:downstream) (Jones et al., 2016).

Another approach that has enabled the control of individual strains in a synthetic consortium is engineering the use of different carbon sources from the different microbial modules. Such an approach, with *E. coli* strains engineered to grow on either glucose or xylose, was used to engineer a two-strain microbial consortium for the production of the flavonoid naringenin. By tuning the concentrations of the two carbon sources, as well as other parameters (inoculation ratio, induction time), the authors developed a process with significantly improved final naringenin titers compared to the monoculture process (Ganesan et al., 2017).

Co-cultivation systems can be exploited for the production of various molecules from simple substrates by employing different downstream strains (Fig. 2C). Additionally, co-cultivation systems allow previously engineered strains to be cultured together without the need of further genetic reconstruction, something that can accelerate process development that is commonly required when additional pathways are inserted in a single cell. For example, Zhang et al. (2015) successfully established a plug-and-play co-cultivation system for prodution of *cis,cis*-muconic acid (MA) and 4-hydroxybenzoic acid (4HB) via a common intermediate 3-dehydroshikimic acid (DHS) by just swapping the downstream strain. Cis,cis-muconic acid was produced, when the upstream DHS precursor provider cell was co-cultivated with downstream MA producer cell while swapping the downstream cell to 4HB producer cell resulted in the production of 4HB (Zhang et al., 2015).

**Fig. 3:**
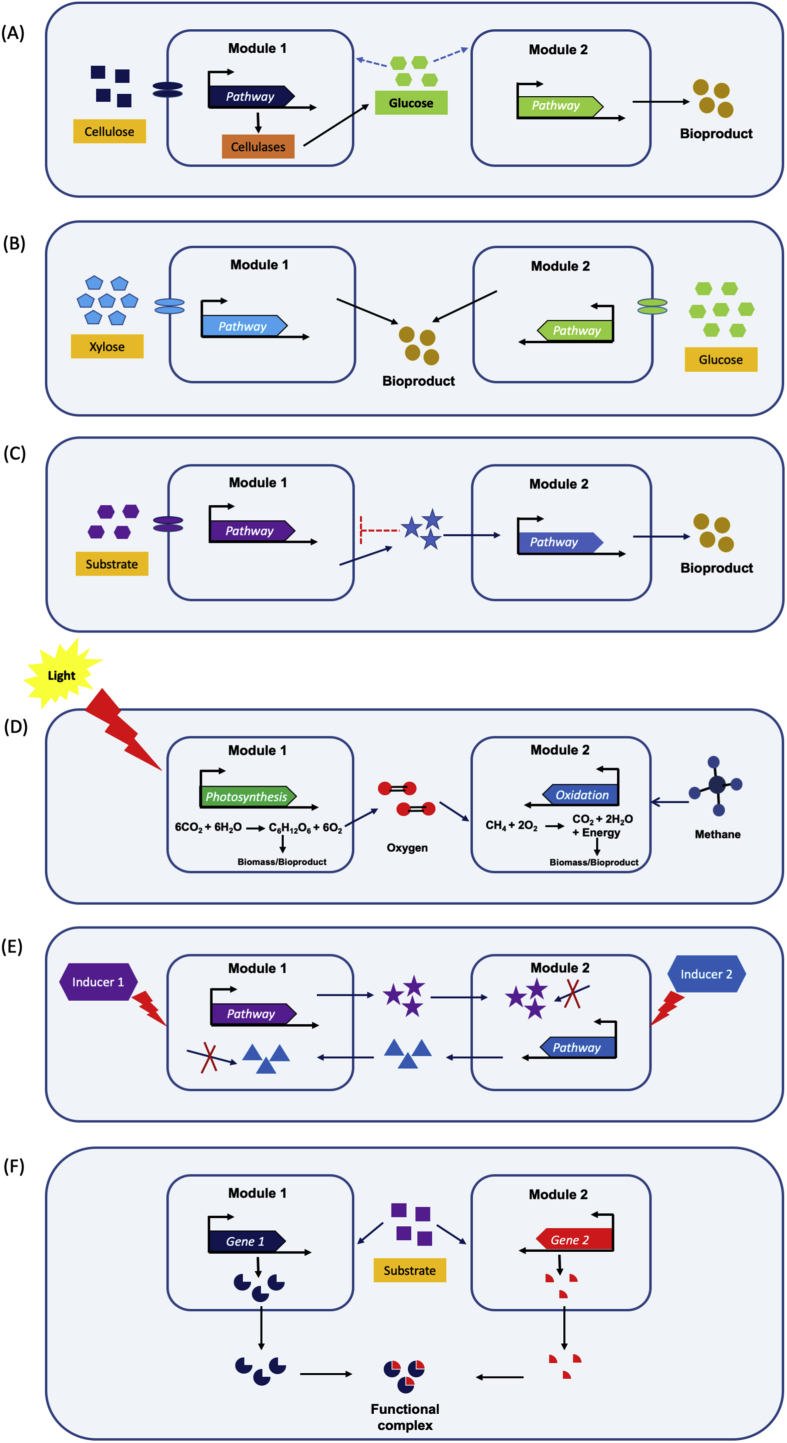
Schematic illustration of different types of co-cultivation systems. (A) Consolidated bioprocess for efficient degradation of lignocellulosic biomass and its utilization, (B) Nutritional divergence to avoid substrate competition between the co-cultivation partners, (C) Cross-feeding in microbial consortium, where one species survives on the side product of the other species while helping the producer to get rid of accumulated toxic side products, (D) Metabolic coupling between oxygenic photosynthesis and methane oxidation to convert greenhouse gasses into microbial biomass (E) Tunable cross-feeding module, where two auxotrophs control each other's growth via inducers. The inducer controls the production of essential metabolites for each partner, which must cross-feed in order to survive in the consortium, and (F) Intercellular complementation, where enzymes secreted out from each constituent strain of the consortium and formed a functional complex.

Lignocellulosic biomass has been considered as a potential raw material for production of various biofuel molecules like ethanol and butanol (Chen, 2011; Lan and Liao, 2013). In a consolidated bioprocess (CBP), simple sugars are first produced from cellulose, which are further converted to bioproducts (Fig. 3A). However, there is no native microorganism available that can simultaneously ferment both glucose and xylose, two major constituent of lignocellulosic biomass hydrolysate (Chen, 2011; Xia et al., 2012). This problem can be addressed by using co-cultivation systems for efficient co-utilization of various substrate mixtures in the same culture medium. One member of the co-cultivation can be engineered to consume xylose while the other constituent member can utilize glucose to make product (Fig. 3B). For example, co-cultivation of two engineered *E. coli* strains, one xylose-selective (glucose deficient) and the other glucose-selective (xylose deficient), utilized xylose and glucose more quickly as compared to a mono-cultivation approach (Eiteman et al., 2008). It was one of the first studies that demonstrated the potential of co-cultivation methods.

Industrial production of natural products has traditionally relied on monocultures because of the more straight-forward metabolic engineering and bioprocess control. However, the accumulation of toxic by-products during fermentation can limit the cell growth and finally lead to decrease in product yield. Co-cultivation allows a symbiotic relationship between the partners in terms of substrate utilization and growth by removal of inhibitory side products, an approach that results in improved biomass and product titers (Fig. 3C). For example, co-cultivation of cellulolytic bacterium *Actinotalea fermentans* and an engineered *Saccharomyces cerevisiae* harbouring methyl halide transferases resulted in the production of methyl-halide from diverse lignocellulosic feedstocks. This symbiotic consortium provides a balance in growth and product formation, where *A. fermentans* ferments cellulose to acetate and ethanol, which is further used by *S. cerevisiae* as a carbon and energy source, preventing accumulation of acetate and ethanol which inhibit the growth of *A. fermentans* (Bayer et al., 2009).

Co-cultivation is also more advantageous compared with two-stage fermentations. Primarily, it decreases the production cost by removing the need of a second sterilization, and decreases the production time, effort and complexity of the fermentation process without compromising the overall yield (Guleria et al., 2016). It also reduces the possibility of contamination during transfer form one bioreactor to another.

Cell-to-cell variation within a population causes significant impact on overall product yield (Wang and Dunlop, 2019; Xiao et al., 2016). There is a report, which showed that in an isogenic free fatty acids producing *E. coli* population, only 15% of total cell population (high producers) yields over half of the total product (Xiao et al., 2016). This heterogeneity in a population is mainly caused by differences in their local environment, genetic variation, and burden of expressing non-native enzyme. Co-cultivation engineering approaches can reduce the risk of these variation by splitting the metabolic load within the population for improved bioproduction (Wang and Dunlop, 2019).

A further advantage of using microbial co-cultivation is the possibility of utilizing cheap substrates such as biomass and organic waste for bioproduction of chemicals and fuels (Sasaki et al., 2018). It has the potential to greatly boost the biotech industry for production of natural products at competitive costs (Bader et al., 2010).

## 3 Microbial consortia in natural systems

In nature, microbes exist in microbial communities composed of many interacting species where they participate in global cycling of oxygen, carbon and nitrogen. In such communities, each member performs chemically difficult tasks to avoid elimination from the consortium. Such naturally occurring microbial consortia have been used for decades in food and other industries (Bader et al., 2010).

A very good example of a natural microbial consortium is of two bioleaching bacteria *Ferroplasma acidiphilum* and *Leptospirillum ferriphilum*, which are always found to coexist in their natural environment i.e. acid mine drainage (Merino et al., 2015). This symbiotic association helps in oxidizing iron and sulfur containing minerals (Merino et al., 2016, 2015). A metabolic model for a mixed culture composed of *L. ferriphilum* and *F. acidiphilum* was reconstructed for deeper understanding of the metabolism of these microorganisms growing together (Merino et al., 2015). Further validation of the model revealed that *F. acidiphilum* utilizes the organic matter secreted by *L. ferriphilum* for growth, maintaining low levels of organic compounds in the culture medium and preventing their toxic effects on *L. ferriphilum* (Merino et al., 2016).

Herbivores’ guts are the natural reservoir of microbial and fungal consortia. These consortia work synergistically to secrete a diverse range of cellulolytic enzymes for degradation of plant biomass to simple sugars efficiently, which are further utilized by the organism itself. There are various reports of synthetic consortia for mimicking the synergisms of natural consortia for efficient degradation of lignocellulosic biomass (Cortes-Tolalpa et al., 2017; Wang et al., 2011). For example, Cortes-Tolalpa et al. (2017) employed 13 different synthetic consortia composed of bacteria and fungi for wheat straw degradation. Out of 13, five showed synergisms and co-cultivation of *Sphingobacterium multivorum* and *Citrobacter freundii* showed maximum synergism i.e. 18.2-fold increase of the produced biomass (Cortes-Tolalpa et al., 2017).

*Lactococcus lactis* naturally evolved into two distinct phenotypic subpopulations when subjected to diauxic shift from glucose to cellobiose. One subpopulation can't metabolize cellobiose (Cel^–^) and stops growing, while the other (Cel^+^) continues to grow by utilizing cellobiose. Cel^–^ population divide faster than Cel^+^ population when galactose was fed as sole carbon source, suggesting bet-hedging phenomenon that helps bacteria adapt against uncertain environmental perturbations (Solopova et al., 2014).

## 4 Recent advances in engineered microbial consortia

Co-cultivation approaches have recently emerged in metabolic engineering, especially for the production of chemicals derived from extensive metabolic pathways (Ganesan et al., 2017; Shong et al., 2012). Table 1 summarizes some recent advances in microbial biosynthesis via co-cultivation. This section elaborates some of the most recent and advanced approaches used in co-cultivation engineering for various products.

**Table 1:** Summary of recent progress in co-cultivation engineering for bioproductions.

Product	Co-cultivation partners	Substrate	Titer	Improvement	Reference
n-Butanol	*E. coli*-*E. coli*	Glucose	5.5 g/L	2-fold	Saini et al. (2015)
Isobutanol	*E. coli- T.reesei*	Cellulosic biomass	1.88 g/L	—	Minty et al. (2013)
2-keto-L-gulonic acid	*G. oxydans* -*K. vulgare*	D-sorbitol	76.6 g/L	29.6%	Wang et al. (2016)
Flavonoid	*E. coli*-*E. coli*	Glycerol and p-coumaric acid	40.77 mg/L	970-fold	Jones et al. (2016)
Muconic acid	*E. coli*-*E. coli*	Glucose and xylose	4.7 g/L	—	Zhang et al. (2015)
Ethanol	*C. phytofermentans -S. cerevisiae*	α-cellulose	22 g/L	2.4-fold	Zuroff et al. (2013)
4-hydroxy benzoic acid	*E. coli*-*E. coli*	Glucose and xylose	2.3 g/L	8.6-fold	Zhang et al. (2015)
3-amino benzoic acid	*E. coli*-*E. coli*	Glucose	48 mg/L	15-fold	Zhang and Stephanopoulos (2016)
Monacolin J	*P. pastoris*- *P. pastoris*	Methanol	593.9 mg/L	55%	Liu et al. (2018)
Lovastatin	*P. pastoris*- *P. pastoris*	Methanol	250.8 mg/L	71%	Liu et al. (2018)
Oxygenated taxanes	*E. coli- S. cerevisiae*	Xylose	33 mg/L	100%	Zhou et al. (2015)
Apigetrin	*E. coli*-*E. coli*	Glucose and p-coumaric acid	16.6 mg/L	2.5 fold	Thuan et al. (2018)

In one example, a new and dynamic co-cultivation technology was developed to convert greenhouse gasses into microbial biomass via oxygenic photosynthesis by employing a methanotrophic bacterium, *Methylomicrobium alcaliphilum* 20z and a cyanobacterium, *Synechococcus* PCC 7002 (Hill et al., 2017) (Fig. 3D). The employment of this interspecies binary consortium provided robust metabolic coupling between oxygenic photosynthesis and methane oxidation. This artificial consortium provided a prototype platform in co-cultivation technology for converting greenhouse gases (GHGs), CH_4_ and CO_2_, into microbial biomass. This system can be customized to produce a range of products along with GHGs remediation.

Rationally engineered co-cultivations have been designed to achieve dynamic interspecies exchange of carbon and energy flow to improve biomass and product formation between *E. coli* and *Acinetobacter baylyi*. *A. baylyi* was made deficient in utilization of glucose by deleting the gluconate permease gene *gntT*. When co-cultivated with *E. coli*, it solely consumed acetate produced from *E. coli* as a side product. This co-cultivation engineering shows how carbon metabolism of these two different species can be connected to remove unwanted side products to improve biomass and product formation (Fig. 3C). (Santala et al., 2014).

Minami et al. (2008) successfully reconstructed the plant alkaloid benzylisoquinoline biosynthetic pathway using microbial and plant enzymes in *E. coli* and *S. cerevisiae*. First *E. coli* cells harboring the reticuline biosynthetic pathway genes were cultured in the presence of dopamine to produce reticuline and later co-cultivated with *S. cerevisiae*, expressing heterologous pathway enzymes to make target alkaloids from reticuline. The resulting co-cultivation was able to produce 7.2 mg/L of magnoflorine within 72 h (Minami et al., 2008).

Industrial production of 2-keto-l-gulonic acid (2-KLG), a vitamin C precursor, is achieved by a two-step fermentation by three strains, *Ketogulonicigenium vulgare, Gluconobacter oxydans* and *Bacillus* spp. (Guleria et al., 2016). In this approach, *G. oxydans* first catalyses the conversion of D-sorbitol to L-sorbose by sorbitol dehydrogenase (SLDH). Next, the whole fermented medium along with other essential substrates is transferred to a second bioreactor and sterilized for the second time. The following second fermentation includes *K. vulgare* and *B. megaterium* which catalyse the conversion of L-sorbose to L-sorbosone by L-sorbose dehydrogenase (SDH, encoded by *sdh*) and its further conversion to 2-KLG by L-sorbone dehydrogenase (SNDH, encoded by *sndh*). Despite the high yield of more than 97%, the long and complex fermentation process remains an engineering conundrum. To address this challenge, a synthetic consortium of *G. oxydans* and *K. vulgare* was reorganized for one-step fermentation of 2-KLG from D-sorbitol. This approach allowed a total yield of 89.7% within 36 h, which was comparable to the conventional two-step fermentation. (Wang et al., 2016). The success of the one-step production process can significantly decrease the cost of vitamin C resulting in a significant impact on the global vitamin market.

Recently, the production of a copolymer, poly(3-hydroxybutyrate-co-3-hydroxyvalerate) (P(3HB-co-3HV) was reported, using a synthetic consortium of *Ralstonia eutropha* (also known as *cupriavidus necator*) and *Bacillus subtilis* from sucrose without precursor feeding (Bhatia et al., 2018). *B. subtilis* hydrolyzes sucrose and also ferments it to propionic acid, which is further utilized by *R. eutropha* to produce (P(3HB-co-3HV). The co-cultivation of *R. eutropha* and *B. subtilis* in optimized media led to the production of (P(3HB-co-3HV) with 66% w/w yield having 16 mol% HV fraction (Bhatia et al., 2018).

Improved production of lovastatin, an anti-hypercholesterolemia pharmaceutical, and its precursor monacolin J was achieved by splitting lovastatin and monacolin J biosynthetic pathways into *P. pastoris* using methanol as an inducer and the sole carbon source. The biosynthetic pathway was segregated and balanced by rationally designing various *P. pastoris*- *P. pastoris* co-cultivation combinations. The synthetic consortium was monitored and controlled with fluorescent reporter protein to achieve balanced growth of the strain modules in a bioreactor. The optimized co-cultivation fermentation in bioreactor yielded 593.9 mg/L monacolin J and 250.8 mg/L lovastatin as compared to 60.0 mg/L monacolin J and 14.4 mg/L lovastatin in mono-cultivation fermentation (Liu et al., 2018).

Co-cultivation engineering approaches have been also employed for production of many biofuel molecules via different metabolic pathways (Kleerebezem and van Loosdrecht, 2007). There are various reports for production of ethanol using co-cultivation methods. Some of them include co-cultivation of immobilized *Z. mobilis* and free cells of *Pichia stipitis* (Fu et al., 2009), co-cultivation of *S. cerevisiae* and *Pachysolen tannophilis* using softwood hydrolysate (Qian et al., 2006), and co-cultivation of restricted catabolite repressed mutant *P. stipitis* and respiratory-deficient *S. cerevisiae* mutant (Kordowska-Wiater and Targoński, 2002). Co-cultivation of free cells of *P. stipites* and immobilized *Zymomonas mobilis* led to ethanol productivity of 1.277 g/l/h with a yield of 0.49–0.50 g/g (Fu et al., 2009).

Similarly, n-butanol was produced by employing two engineered *E. coli* strains, a butyrate-producing strain as upstream strain and a butyrate conversion strain as downstream strain. The upstream strain harboured genes *phaA*, *hbd*, *crt*, *ter* and *atoDA* for biosynthesis of butyrate from glucose while the downstream strain harboured endogenous gene *atoDA* and *Clostridium* gene *adhE2*. The atoDA facilitated interconversion of butyrate and butyryl-CoA via acetate, which freely cross the cell membrane. The butyrate conversion strain converted butyrate to butanol and released acetate, which further recycled by the butyrate producing strain. When both strains co-cultivated, 5.5 g/L of n-butanol was produced from glucose in 24 h, which was 2-fold higher than that of reference strain produced during mono-cultivation under the same conditions (Saini et al., 2015). Further improvement in butanol production by using co-cultivation system was achieved by using a symbiotic consortium of *C. acetobutylicum* TSH1 carrying deletion of *maf* gene, and *Bacillus cereus* TSH2. This synthetic consortium resulted in the production of 13.9 ± 1.0 g/L of butanol (Mi et al., 2018).

Co-cultivations have been widely studied in consolidated bioprocessing (CBP) for production of different biofuels such as ethanol, butanol and hydrogen (Jiang et al., 2018; Wang et al., 2015). CBP via microbial consortium allows efficient biomass degradation as well as substrate utilization by individual strains for the production of different bioproducts (Fig. 3A). Some examples include; (i) Production of biohydrogen from cellulose using a microbial consortium of *Clostridium thermocellum* DSM 1237, a cellulolytic bacterium and *Clostridium thermopalmarium* DSM 5974, a non-cellulolytic hydrogen producing bacterium (Geng et al., 2010). (ii) Production of butyric acid from sucrose using co-cultivation of *Bacillus* sp. SGP1 and *Clostridium tyrobutyricum* (Dwidar et al., 2013). (iii) Co-cultivation of two *E. coli* strains, a carbohydrate catabolite repression (CCR) insensitive glucose-selective strain and a xylose-selective strain, that efficiently co-utilized both sugars and produced 5.2 g/L n-butanol at 63% of the theoretical yield (Saini et al., 2017). Similarly, biproduction of hydrogen was established by co-cultivation of *Clostridium thermocellum* and *Thermoanaerobacterium aotearoense* from pretreated sugarcane bagasse (SCB). A titer of 50.05 ± 1.51 mmol/L hydrogen was achieved with 4% pretreated SCB at 55 °C (Cheng and Zhu, 2013), which was further improved by supplementation of CaCO_3_ to reach a final titer of 87.56 ± 4.08 mmol/L from 2% pretreated SCB with a yield of 4.38 mmol H2/g SCB (Bu et al., 2017).

Flavonoids are high-value compounds with important nutraceutical and pharmaceutical applications. Synthesis of flavonoids requires different pathway-dependent cofactors and precursors, which need to be balanced in order to achieve efficient yield. In order to achieve high titers and yields, different co-cultivation approaches have been applied for their production. In one approach, the biosynthetic pathway containing six genes was split into two modules, each comprising three genes, as per requirement of co-factor i.e. malonyl-CoA and NADPH. This strategy improved flavan-3-ol production to 40.7 mg/L, a 970-fold improvement over a previous report using mono-cultivation system (Chemler et al., 2007; Jones et al., 2016). In a second approach, a synthetic consortium containing four *E. coli* strains was established, which collectively expressed 15 pathway genes from different plants and microbes for production of anthocyanins (Jones et al., 2017). The combination of four engineered strains resulted in the production of anthocyanins directly from glucose for the first time (Jones et al., 2017). This was the first implementation of a polyculture consortium comprising 4 engineered strains and resulted in the de novo biosynthesis of anthocyanins for the first time.

An example of cross-feeding is a microbial consortium consisting of *Citrobacter amalonaticus* Y19 and *Sporomusa ovata* that has been used for production of acetic acid from carbon monoxide (CO) as the sole carbon source. *C. amalonaticus* Y19 produced CO_2_ and H_2_ from water-gas shift reaction which were further utilized by *S. ovata*. The production of acetate from CO was 1.47 mM, 0.807 mM, and negligible in the co-cultivation, mono-cultivation of *S. ovata*, and mono-cultivation of *C. amalonaticus*, respectively. This syntrophic cooperation can be further utilized for production of various biofuel molecules using CO as carbon source to help address environmental problems (Lee et al., 2018). In an another example, two *E. coli* auxotrophs were constructed to cross-feed tryptophan and tyrosine, which enables continuous tuning of the growth rate and composition of the consortium (Fig. 3E) (Kerner et al., 2012).

Moreover, co-cultivation systems have been employed for enhanced degradation of different pesticides. There is a report of degradation of β-cypermethrin (β-CY) and 3-phenoxybenzoic acid (3-PBA) by co-cultivation of *Bacillus licheniformis* B-1 and *Aspergillus oryzae* M-4 (Zhao et al., 2016). Co-cultivation strategy has been also demonstrated for efficient degradation of paracetamol up to concentrations of 4 g/L by microbial consortium containing three strains (Zhang et al., 2013).

In order to elucidate the interaction within the microbial communities Niehaus et al. (2018) constructed a mechanistic model framework, in which microbial chemical mediators were incorporated in order to elucidate how microbial species interact in coexistence. The model shows that growth facilitation and self-restraint interactions played a key role in assembling communities. They found that facilitation (i.e. stimulation of growth of other community members) is favored in coexistent communities, whereas inhibition of other species (but not self) is disfavored. They also observed that in many instances, these effects are causal, that is, facilitation and self-restraint (i.e. inhibition of self) interactions encourage coexistence, but inhibitory interactions that suppress other species are detrimental to coexistence (Niehaus et al., 2018).

Co-cultivation approaches has been successfully implemented for synthesis of functional minicellulosomes (an enzyme complex) (Arai et al., 2007; Goyal et al., 2011). In one example, when a *B. subtilis* expressing *Clostridium cellulovorans* gene *minicbpA* was co-cultivated with a *B. subtilis* strain expressing either an endoglucanase or a xylanase, it resulted in minicellulosome formation with both miniCbpA and the cellulosomal activity. They called this phenomenon ‘‘intercellular complementation’’ as both miniCbpA and the cellulosomal enzymes secreted out and formed a functional minicellulosome (Arai et al., 2007) (Fig. 3F).

Recently, Xiu et al. (2017) developed an RNA riboswitch-based biosensor module having dual fluorescence reporters for rapid screening of naringenin overproducing *E. coli* strains in co-culture using flow cytometry. Naringenin acted as an inducer for reporter gene activation which led to increase in fluorescent signal generation, while in the absence of naringenin, the expression of reporter gene was prevented by formation of a three dimensional structure of the aptamer mRNA. This is the first report of a producer-biosensor co-culture system that has been utilized for *in vivo* product quantification. This strategy can be applied for real-time measurement of intracellular or extracellular metabolites (Xiu et al., 2017).

## 5 Challenges and limitations

Despite many advantages over monocultures, a number of challenges remain when using co-cultivation approaches for production purposes.

### 5.1 Co-cultivation compatibility

Strain compatibility is a key factor in any successful co-cultivation system. The co-cultivation constituent stains must be able to grow efficiently in the same growth parameters, such as media, pH, temperature and oxygen requirement and must not produce toxic compounds that significantly harm the other members of the microbial community (Zhang and Wang, 2016). These criteria can be addressed by employing microbial strains derived from the same species (Fig. 1A), as they require similar growth conditions and possess similar growth rates. However, problems arise when multiple strains form different species are used for constructing the synthetic microbial consortium, as the growth rates of different species vary to a large extent and different species have different media requirements. For these reasons, one species can dominate and take over the culture during co-cultivation, which leads to disruption of the ratio of the participating microbial species and finally in poor production yields. In order to address this problem, one possibility is to introduce positive interactions between the microbial partners during the co-cultivation fermentation.

### 5.2 Substrate competition

If co-cultivation partners utilize the same growth resources, it will result in competitive exclusion and unstable co-cultivation that is undesirable for industrial fermentation process for bioproduction. This problem has been addressed by using either syntrophy or nutritional divergence approaches in co-cultivation systems. These strategies help in making dynamic and symbiotic microbial interactions within the consortium by efficient carbon channelling and energy flow. However, all organisms have their own nutritional requirements and preferences, which make it difficult to apply this approach. Therefore, a cross-feeding or nutritional divergence within a co-cultivation is desirable, which allows reduction or elimination of a microbial species from the consortium, and make coexistence possible (Fig. 3B, C and 3E).

### 5.3 Reproducibility

Balancing the population ratio in microbial consortia at desired values throughout the co-cultivation process is the major bottleneck for bioproduction. Co-cultivation population composition can fluctuate to a large extent due to various factors such as differences in doubling time, substrate competition and toxic by-products produced by consortium members (Zhou et al., 2015). It greatly impacts the reproducibility of co-cultivation engineering studies. Reactor volume can also affect co-cultivation viability (Shou et al., 2007). The stability of the culture population ratio decreases while increasing the culture volume, potentially leading to heterogeneity within the system. However, there are certain ways to stabilize the strain-to-strain ratios between the co-cultivation members so that one strain does not eliminate the other. Fine tuning of inoculation ratio between co-cultivation partners greatly affects overall production, although the sub-population ratio is often found to fluctuate or change during the cultivation period (Jones and Koffas, 2016; Li et al., 2019). In addition, mutualistic growth has also been studied for maintaining desired population composition of the engineered co-cultivations (Kerner et al., 2012).

Moreover, there are tools based on quorum sensing that are being developed to manipulate growth rate and biomass through cell-to-cell communication (Carbonell et al., 2002). This provides a promising way to control growth and metabolic pathway coordination between the co-cultivation members. Harcombe et al. (2014) developed a computer model termed COMETS, that computes the internal metabolic budget of the cell involving thousands of reactions and predicts how fast a microbe can grow in the community. They applied this model to a three-member consortium that incorporates *Methylobacterium extorquens* AM1 into the *E. coli/S. enterica* co-culture based on tuneable symbiosis (Harcombe et al., 2014).

### 5.4 Exchange of metabolites

Transportation of intermediate pathway metabolites between the different strains participating in an engineered consortium to produce final product is a major limitation in co-cultivation systems as varied range of pathway intermediates, such as various CoA species and phosphorylated molecules, have limited mobility for exchange across the cell membrane and it is difficult to engineer transport systems specifically for such compounds. Keeping this in mind, pathway module should be carefully segregated between the constituent strains so that the linking metabolite can easily be transported between the co-cultivation members. Moreover, membrane transporters can be engineered to efflux the pathway intermediates in the desired direction (Zhou et al., 2012).

### 5.5 Data acquisition

Acquisition of comprehensive data for co-cultivations holds a great challenge as industrial, medical and environmental applications require in-depth data collection and characterization (El-Ali et al., 2006; Goers et al., 2014). Determining metabolic flux distribution in a co-cultivation system is much easier than within cells, giving an edge over mono-cultivation systems for obtaining insights into metabolism (Rollié et al., 2012). However, the metabolic interaction within the artificial consortium is difficult to elucidate, as microbial members may exchange more than their known interacting metabolites (Chuang et al., 2010; Sabra et al., 2010; Schmidt et al., 2011). Nevertheless, there are few reports of complete experimental and theoretical strategies for co-cultivation characterization, which determined metabolic flux distributions between co-cultivation species simultaneously without the need for physical separation of cells (Gebreselassie and Antoniewicz, 2015).

## 6 Conclusion and future perspectives

Microbial biosynthesis via co-cultivation engineering provides a paradigm shift in metabolic pathway balancing. It broadens the possibilities to tune complex metabolic pathways and can be customized for efficient production of a variety of bioproducts. Co-cultivation engineering has several advantages over mono-cultivation systems such as robustness, modularity, higher tolerance (toxic intermediate/waste produced from one partner get consumed/degraded by the other partner) and higher productivity. It utilizes the metabolic power and resources of each co-cultivation partner to meet the demand of specific co-factors and precursors and thus improves the conversion yield of the modularized biosynthetic pathway. This approach allows to produce more complex compounds with improved productivity by distributing the metabolic pathway between each consortium member. Co-cultivation fermentations may lead to enhanced production performance, and allow the utilization of cheaper substrates. Moreover, artificial consortia open the door to address the issues of functional expression of complex biosynthetic pathway enzymes without compromising the yield and product quality. They can also reduce the effort of reconstitution of recombinant biosynthetic pathways.

As an emerging research area in the field of metabolic engineering, co-cultivation engineering is still in its infancy. Most of the recent reports on co-cultivation engineering that are based on employment of microbial consortia have only two constituent strains/species in order to achieve their engineering goals. Co-cultivation of multiple populations is more complicated, as co-cultivation behaviour of individual strains using common cultivation methods is still unknown and potentially more challenging to control when increasing the number of the constituent strains/species. However, recent development in co-cultivation engineering has greatly expanded our understanding of microbial behaviour in communities (Wang et al., 2016; Zuñiga et al., 2017).

While the potential of synthetic microbial consortia holds great promise, there are inherent challenges that need to be addressed with the help of synthetic biology approaches. It is anticipated that co-cultivations comprising multiple specialized members, or polycultures, will be developed and utilized for meeting the demand of more complicated biosynthetic pathways in the near future.
